# Preserved fractal character of structural brain networks is associated with covert consciousness after severe brain injury

**DOI:** 10.1016/j.nicl.2021.102682

**Published:** 2021-04-21

**Authors:** Andrea I. Luppi, Michael M. Craig, Peter Coppola, Alexander R.D. Peattie, Paola Finoia, Guy B. Williams, Judith Allanson, John D. Pickard, David K. Menon, Emmanuel A. Stamatakis

**Affiliations:** a Division of Anaesthesia, School of Clinical Medicine, University of Cambridge, Addenbrooke's Hospital, Hills Rd, CB2 0SP, Cambridge, United Kingdom; b Department of Clinical Neurosciences, School of Clinical Medicine, University of Cambridge, Addenbrooke's Hospital, Hills Rd, CB2 0SP, Cambridge, United Kingdom; c Division of Neurosurgery, School of Clinical Medicine, University of Cambridge, Addenbrooke's Hospital, Hills Rd, CB2 0SP, Cambridge, United Kingdom; d Wolfson Brain Imaging Centre, University of Cambridge, Cambridge Biomedical Campus (Box 65), Cambridge CB2 0QQ, United Kingdom; e Department of Neurosciences, Cambridge University Hospitals NHS Foundation, Addenbrooke's Hospital, Hills Rd, CB2 0SP, Cambridge, United Kingdom

**Keywords:** Disorders of consciousness, Brain injury, Diffusion MRI, Fractal, Brain network, Cognitive-motor dissociation

## Abstract

Self-similarity is ubiquitous throughout natural phenomena, including the human brain. Recent evidence indicates that fractal dimension of functional brain networks, a measure of self-similarity, is diminished in patients diagnosed with disorders of consciousness arising from severe brain injury. Here, we set out to investigate whether loss of self-similarity is observed in the structural connectome of patients with disorders of consciousness. Using diffusion MRI tractography from N = 11 patients in a minimally conscious state (MCS), N = 10 patients diagnosed with unresponsive wakefulness syndrome (UWS), and N = 20 healthy controls, we show that fractal dimension of structural brain networks is diminished in DOC patients. Remarkably, we also show that fractal dimension of structural brain networks is preserved in patients who exhibit evidence of covert consciousness by performing mental imagery tasks during functional MRI scanning. These results demonstrate that differences in fractal dimension of structural brain networks are quantitatively associated with chronic loss of consciousness induced by severe brain injury, highlighting the close connection between structural organisation of the human brain and its ability to support cognitive function.

## Highlights

• We study structural brain networks in patients with disorders of consciousness (DOC)
• Structural brain networks are less fractal (self-similar) in patients than controls.
• Preserved fractal dimension is associated with covert consciousness in DOC patients.

## 1 Introduction

Self-similarity is ubiquitous throughout natural phenomena – from the progressively branching structure of trees, rivers and blood vessels, to the human brain being a network composed of nested sub-networks. Self-similarity across scales can be quantified in terms of fractal dimension, the extent that the same organisation can be observed across levels of detail, i.e. when considering the whole system or “zooming in” on its parts. Importantly, it has been argued that self-similar (fractal) organisation may be the key property that enables the human brain to balance its competing needs for functional integration and differentiation, whereby a backbone of self-similar modules is turned into a “small world” by the addition of weaker connections (Gallos et al., 2012, Gallos et al., 2012b).

Indeed, previous research has revealed the neurobiological relevance of variations in anatomical and functional fractal dimension. Anatomically, fractal dimension of grey and white matter structures has been associated with cognitive outcomes – not only in the healthy brain, but also in aging or disease (Im et al., 2006, King et al., 2009, Mustafa et al., 2012, Tae et al., 2005). Functionally, electroencephalography (EEG) and functional MRI can provide estimates of fractal dimension in both space and time, which have been shown to reflect cognitive performance (Bornas et al., 2013), the altered state of consciousness induced by psychedelics (Varley et al., 2020a) and also loss of consciousness due to natural sleep or different anaesthetics (Ruiz de Miras et al., 2019, Varley et al., 2020c).

Crucially, fractal dimension of functional brain networks has recently been shown to distinguish between disorders of consciousness (DOC) of different severity, whether arising from anoxic or traumatic brain injury (Varley et al., 2020b). This evidence is especially relevant because fractal dimension can be viewed as quantifying a system’s complexity: it has been shown to correlate with other measures of spatial and temporal complexity, such as entropy and compressibility (Chen, 2020, Varley et al., 2020c). Importantly, complexity is increasingly recognised as a fundamental requirement for the brain’s ability to support a wide variety of conscious states (Carhart-Harris, 2018, Carhart-Harris et al., 2014, Luppi et al., 2020a, Tononi et al., 1994).

To date, investigations of fractal dimension in states where consciousness is diminished or lost (e.g. sleep, anaesthesia, DOC) have focused on functional neuroimaging signals (Klonowski et al., 2005, Ruiz de Miras et al., 2019, Spasic et al., 2011, Varley et al., 2020c, Varley et al., 2020b). Whereas no major reorganisation of brain structure is expected to occur as a result of the temporary loss of consciousness induced by sleep or anaesthesia, disorders of consciousness typically involve severe brain injury, including reorganisation of white matter fibers and their network organisation (Cavaliere et al., 2015, Fernández-Espejo et al., 2012, Fernández-Espejo et al., 2011, Kuceyeski et al., 2016, Lant et al., 2016, Newcombe et al., 2010, Wang et al., 2018, Weng et al., 2017, Wu et al., 2018, Zheng et al., 2017).

Structural brain networks comprise white matter fibers providing physical connections between brain regions (the “connectome”) (Hagmann et al., 2008, Sporns et al., 2005), which constitute the scaffolding for communication of information across the brain (Bettinardi et al., 2017, Deco and Jirsa, 2012). By exploiting anisotropic diffusion of water molecules along axonal bundles, diffusion-weighted magnetic resonance imaging (DWI) enables the study of white matter pathways in the brain in vivo and non-invasively (Le Bihan and Johansen-Berg, 2012). Structural networks are also less susceptible than functional ones to confounds such as momentary arousal levels, and tend to exhibit higher reproducibility within individuals (Lawrence et al., 2018), which may provide additional prognostic value. However, it is presently unknown whether fractal dimension of structural brain networks is altered in patients suffering from DOCs as a result of severe brain injury.

**Fig. 1:**
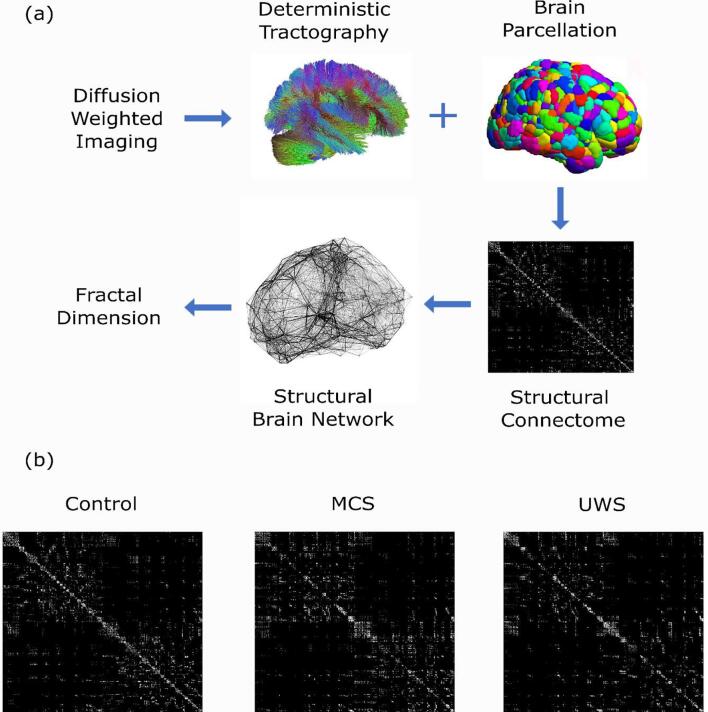
Overview of the analysis. (a) Data processing pipeline for each individual. After reconstruction and deterministic tractography of the DWI data, the Schaefer scale 1000 parcellation (Schaefer et al., 2018) was applied to obtain a connectivity matrix indicating the number of white matter streamlines between each pair of cortical regions (representing the individual’s structural connectome); a structural brain network was then constructed, and finally its fractal dimension was computed. (b) Three 1000 × 1000 adjacency matrices, representing the structural connectome of representative individuals from the healthy control group, the MCS group, and the UWS group.

To address this question, here we compared the fractal dimension of structural brain networks obtained from DWI data of N = 20 healthy controls, N = 11 patients diagnosed as being in a minimally conscious state (MCS), and N = 10 patients suffering from unresponsive wakefulness syndrome/vegetative state (UWS), who had lost consciousness chronically after severe hypoxic or traumatic brain injury (Fig. 1). Although previous investigations of fractal dimension of brain networks required the networks to be binarized (Varley et al., 2020b), we capitalised on a recently developed measure of fractal dimension for weighted networks, which allows edge weights (here, number of white matter streamlines between brain regions) to be taken into account, without discarding this potentially valuable information (Wei et al., 2013). Our aim was to investigate whether the structural brain networks of patients with chronic DOCs exhibit reduced fractal dimension, and whether it differs based on diagnostic category (MCS vs UWS). Finally, we sought to determine whether preserved fractal dimension of structural brain networks is associated with the DOC patients’ ability to provide evidence of covert consciousness, by capitalising on the availability of task-based functional MRI data from the same cohort of DOC patients.

## 2 Materials and methods

### 2.1 Ethics statement

All clinical investigations were conducted in accordance with the Declaration of Helsinki, and ethical approval for this study was provided by the National Research Ethics Service (National Health Service, UK; LREC reference 99/391).

### 2.2 Patients with disorders of consciousness

A sample of 71 DOC patients with chronic disorders of consciousness were recruited from specialised long-term care centres between January 2010 and December 2015. Patients from this cohort have been studied before, in the context of functional MRI analyses (Luppi et al., 2020b, Luppi et al., 2019, Varley et al., 2020b). To be considered for inclusion in the study, patients must have been diagnosed with a disorder of consciousness, and written informed consent was obtained from their legal representative. Patients were excluded from participation if they could not be transported to Addenbrooke’s Hospital (Cambridge, UK), if any medical condition made it unsafe for the patient to participate (this decision was made by clinical personnel blinded to the specific aims of the study), or if any reason made the patient unsuitable to enter the MRI scanner environment (e.g. non-MRI-safe implants). Further exclusion criteria were significant pre-existing mental health problems, or insufficient fluency in the English language prior to injury. Patients spent a total of five days (including arrival and departure days) at Addenbrooke's Hospital, where each of them underwent clinical and neuroimaging testing. Brain scanning took place at the Wolfson Brain Imaging Centre (Addenbrooke’s Hospital, Cambridge, UK); medication prescribed to each patient was maintained during scanning.

Since this study focused on whole-brain structural networks, coverage of most of the brain was required, and we followed the same criteria as in our previous studies (Luppi et al., 2020b, Luppi et al., 2019) whereby before analysis took place, patients were systematically excluded if an expert neuroanatomist blinded to diagnosis judged that they displayed excessive focal brain damage (over one third of one hemisphere), or if brain damage led to suboptimal segmentation and normalisation, or if patients displayed excessive head motion in the scanner (defined as more than 3 mm translation or 3 degrees rotation). One additional patient was excluded due to incomplete acquisition of diffusion-weighted images. A total of 21 adults (13 males; 17–70 years; mean time post injury: 13 months) meeting diagnostic criteria for unresponsive wakefulness syndrome/vegetative state (UWS; N = 10) or minimally conscious state (MCS; N = 11) due to brain injury were included in this study (Table 1).

**Table 1:** Demographic and clinical information for patients with Disorders of Consciousness.

Sex	Age	Months post injury	Aetiology	Diagnosis	CRS-R Score	Tennis	Spat Nav	Classification	Scan
M	46	23	TBI	UWS	6	no evidence	no evidence	FMRI-	12 dir
M	57	14	TBI	MCS	12	no evidence	no evidence	FMRI-	12 dir
M	35	34	Anoxic	UWS	8	no evidence	no evidence	FMRI-	12 dir
M	17	17	Anoxic	UWS	8	no evidence	positive	FMRI+	12 dir
F	31	9	Anoxic	MCS	10	no evidence	no evidence	FMRI-	12 dir
F	38	13	TBI	MCS	11	positive	no evidence	FMRI+	12 dir
M	29	68	TBI	MCS	10	SMA+ ve	PPA+ ve	FMRI+	63 dir
M	23	4	TBI	MCS	7	SMA+ ve	no evidence	FMRI+	63 dir
F	70	11	Cerebral bleed	MCS	9	no evidence	no evidence	FMRI-	63 dir
F	30	6	Anoxic	MCS	9	PMC+ ve	no evidence	FMRI+	63 dir
F	36	6	Anoxic	UWS	8	no evidence	PPA+ ve	FMRI+	63 dir
M	22	5	Anoxic	UWS	7	no evidence	no evidence	FMRI-	63 dir
M	40	14	Anoxic	UWS	7	no evidence	no evidence	FMRI-	63 dir
F	62	7	Anoxic	UWS	7	no evidence	no evidence	FMRI-	63 dir
M	46	10	Anoxic	UWS	5	no evidence	no evidence	FMRI-	63 dir
M	21	7	TBI	MCS	11	no evidence	no evidence	FMRI-	63 dir
M	67	14	TBI	MCS	11	SMA+ ve	PPA+ ve	FMRI+	63 dir
F	55	6	Hypoxia	UWS	7	no evidence	no evidence	FMRI-	63 dir
M	28	14	TBI	MCS	8	positive	positive	FMRI+	63 dir
M	22	12	TBI	MCS	10	no evidence	no evidence	FMRI-	63 dir
F	28	8	ADEM	UWS	6	no evidence	no evidence	FMRI-	63 dir

CRS-R, Coma Recovery Scale-Revised, obtained closest to the scan; UWS, Unresponsive Wakefulness Syndrome; MCS, Minimally Conscious State; TBI, Traumatic Brain Injury; FMRI-, negative responders to mental imagery tasks; FMRI+, positive responders to either mental imagery task; SMA, supplementary motor area; PPA, parahippocampal place area; PMC, pre-motor cortex.

#### 2.2.1 Clinical assessment

Coma Recovery Scale-Revised (CRS-R) assessments were recorded at least daily for the five days of admission. If behavioural responses were not indicative of awareness at any time, patients were classified as UWS. In contrast, patients who exhibited simple automatic motor reactions (e.g., scratching, pulling the bed sheet), visual fixation and pursuit, or localisation to noxious stimulation, were classified as being in a minimally conscious state (MCS) (note that due to the limited size of our sample of MCS patients, we do not sub-divide these patients into MCS- and MCS+) (Bruno et al., 2011, Wannez et al., 2018) (Table 1).

#### 2.2.2 Identification of responsiveness to mental imagery tasks

In addition to clinical diagnosis, which is based on overt behavioural responsiveness, we also stratified patients in two groups (FMRI+ and FMRI-) based on their ability to perform mental imagery tasks on command during fMRI scanning (Luppi et al., 2020b, Craig et al., 2021), using an established methodology that has previously been applied to DOC patients and healthy individuals (Fernández-Espejo et al., 2014, Monti et al., 2010, Owen et al., 2006). The rationale for this approach is that some patients may fail to exhibit overt behavioural responses due to motor impairments rather than because they are unconscious, and responsiveness to fMRI tasks can reveal this “covert consciousness” – although of course it is important to note that absence of fMRI responsiveness does not constitute conclusive evidence of unconsciousness (MacDonald et al., 2015).

The first mental imagery task (referred to as the “tennis task”) involved motor imagery, whereby each patient was asked to “imagine being on a tennis court swinging their arm to hit the ball back and forth with an imagined opponent” (Luppi et al., 2020b, Monti et al., 2010, Owen et al., 2006). The second mental imagery task (referred to as the “navigation task”) involved spatial imagery, whereby the patient was required to imagine walking around the rooms of their house, or the streets of a familiar city, and to visualise what they would see if they were there (Luppi et al., 2020b, Craig et al., 2021). Each task was structured to comprise five cycles, alternating 30 s of imagery and 30 s of rest, during which patients were instructed to just stay still with eyes closed. Each block of mental imagery was cued with the spoken word “tennis” or “navigation”, respectively, whereas the rest blocks were cued with the word “relax”.

For each patient, classification into FMRI+ and FMRI- groups was based on the results of univariate fMRI analysis conducted on both the motor and spatial mental imagery tasks (using FSL version 5.0.9; https://fsl.fmrib.ox.ac.uk/fsl/fslwiki/). For each functional scan, a general linear model was used to contrast periods of rest and active imagery (Monti et al., 2010).

If a patient’s fMRI activation was significantly greater than rest in task-relevant regions (cluster-forming voxel-level threshold of z > 2.3, cluster-corrected *p* < 0.05) during either or both of the two mental imagery tasks, this was taken as evidence that the patient was performing the task (and therefore responding to command), and the patient was classified as FMRI+, constituting evidence of covert consciousness. We identified N = 8 such FMRI+ patients. Conversely, N = 13 patients did not respond to either task, and therefore failed to provide evidence of covert consciousness; we designated these patients as “FMRI-” (Craig et al., 2021) (Table 1).

### 2.3 Healthy controls

We also acquired diffusion MRI data from N = 20 healthy volunteers (13 males; 19–57 years), with no history of psychiatric or neurological disorders. The mean age was not significantly different between healthy controls (M = 35.75; SD = 11.42) and DOC patients (M = 38.24; SD = 15.96) (*t*(39) = −0.57, *p* = 0.571, Hedges’s *g* = −0.18; permutation-based *t*-test).

### 2.4 Acquisition of diffusion-weighted data

As the patients’ data were acquired over the course of several years, two different diffusion-weighted image acquisition schemes were used. The first acquisition scheme (used for the N = 6 patients whose data were acquired earliest in time) used an echo planar sequence (TR = 8300 ms, TE = 98 ms, matrix size = 96 × 96, 63 slices, slice thickness = 2 mm, no gap, flip angle = 90 degrees). This included diffusion sensitising gradients applied along 12 non-collinear directions with 5b-values that ranged from 340 to 1590 s/mm2 and 5b = 0 images (Correia et al., 2009). The more recent acquisition scheme (used for the more recently scanned patients, and for all healthy controls) instead involved the use of 63 directions with a b-value of 1000 s/mm2. Both DWI acquisition types have been used before in the context of structural connectivity analysis in DOC patients (Wang et al., 2018, Zheng et al., 2017); note that none of the four patient sub-groups considered here (MCS, UWS, FMRI+ and FMRI-) was exclusively made up of patients whose data had been acquired in one of the two sequences (Table 1). Nevertheless, to account for this potential confound, we also included acquisition type as a covariate of no interest.

### 2.5 DWI preprocessing

The diffusion data were preprocessed with MRtrix3 tools (Tournier et al., 2019). After manually removing diffusion-weighted volumes with substantial distortion (Zheng et al., 2017), the pipeline involved the following steps: (i) DWI data denoising by exploiting data redundancy in the PCA domain (Veraart et al., 2016) (*dwidenoise* command); (ii) Correction for distortions induced by eddy currents and subject motion by registering all DWIs to b0, using FSL’s *eddy* tool (through MRtrix3 *dwipreproc* command); (iii) rotation of the diffusion gradient vectors to account for subject motion estimated by *eddy* (Leemans and Jones, 2009)*;* (iv) b1 field inhomogeneity correction for DWI volumes (*dwibiascorrect* command); (v) generation of a brain mask through a combination of MRtrix3 *dwi2mask* and FSL *BET* commands. The number of motion-corrupted volumes was significantly different between healthy controls (mean = 0) and DOC patients (mean = 3.5 ±3.8), t(39) = -4.13, p < 0.001; therefore, the number of removed volumes was included as a covariate of no interest in our analysis.

### 2.6 DTI data reconstruction and fiber tracking

DTI data were reconstructed from the preprocessed DWIs using DSI Studio (www.dsi-studio.labsolver.org), which implements the model-free q-space diffeomorphic reconstruction (QSDR (Yeh et al., 2011)).

QSDR is especially well-suited for comparisons between groups (Tan et al., 2019, Yeh et al., 2013, 2011), by reconstructing the distribution of the density of diffusing water in standard space, thereby preserving the continuity of fiber geometry for subsequent tracking (Yeh et al., 2011). Indeed, QSDR has successfully been used to investigate structural networks in healthy individuals (Gu et al., 2015) as well as brain-injured patients (Gu et al., 2017), including patients with disorders of consciousness (Tan et al., 2019b). After reconstructing diffusion-weighted images in each subject’s native space, QSDR computes values of quantitative anisotropy (QA) in each voxel, which are used to nonlinearly warp the brain to DSI Studio’s template QA volume in Montreal Neurological Institute (MNI) space. Spin density functions (SDFs) were then reconstructed in the standard space, using a mean diffusion distance of 1.25 mm with three fiber orientations per voxel (Yeh et al., 2011).

After QSDR reconstruction, 1,000,000 streamlines between brain regions were tracked with DSI Studio’s high-performing “FACT” deterministic algorithm. We adopted previously established parameters (Gu et al., 2015, Luppi and Stamatakis, 2021): angular cutoff = 55◦, step size = 1.0 mm, tract length between 10 mm (minimum) and 400 mm (maximum), no spin density function smoothing, and QA threshold determined by DWI signal in the cerebro-spinal fluid. Streamlines with improper termination locations were automatically screened by DSI Studio’s algorithm using a white matter mask, obtained by applying a default anisotropy threshold of 0.6 Otsu's threshold to the anisotropy values of the spin density function (Gu et al., 2015, Luppi and Stamatakis, 2021, Medaglia et al., 2016).

### 2.7 Structural network construction

A network consists of two basic elements: nodes, and the edges connecting them. To construct structural brain networks, we computed the number of streamlines between each pair of 1000 cortical regions of interest (ROIs), derived from the largest scale of the Schaefer multi-scale atlas (Schaefer et al., 2018). The Schaefer-1000 atlas has been used in recent analyses of brain fractal dimension in healthy individuals (Varley et al., 2020a) and also DOC patients (Varley et al., 2020b). Nevertheless, to demonstrate the robustness of our analysis to atlas choice, we used the largest scale of the Lausanne atlas, with the same number of cortical ROIs (Cammoun et al., 2012). For this analysis, the Lausanne parcels were dilated by 2 voxels to extend them to the grey-matter-white matter interface, following previous tractography work using the same parcellation (Gu et al., 2015, Medaglia et al., 2016). We also replicated our results using a smaller scale of the Schaefer atlas with 400 ROIs, to demonstrate their robustness to parcellation size. As further validation analysis, we also employed a different tractography procedure, whereby probabilistic tractography implemented in the MRtrix3 toolbox (Tournier et al., 2019) was used to track 1,000,000 streamlines in each subject’s native space, and subsequently FSL’s *flirt* tool was used to bring the MNI-space Schaefer-1000 cortical parcellation (Schaefer et al., 2018) into each subject’s native space using an affine transformation (rather than a nonlinear one, as applied by DSI Studio), to quantify the number of streamlines between each pair of regions in native space.

ROIs represent the nodes of the brain network. Then, edges between nodes were selected using the Efficiency Cost Optimisation (ECO) criterion, which is designed to optimise the trade-off function *J* between wiring cost ρ and overall network efficiency (i.e. the sum of global efficiency $E_{g}$and mean local efficiency $E_{l}$of the network) (De Vico Fallani et al., 2017):

(1) $$J=\frac{E_{g}+E_{l}}{\rho }$$

Because it highlights the network’s topological organisation, ECO has excellent ability to discriminate between groups with different network topologies, including controls and patients, across different datasets and imaging modalities (MRI, EEG) (De Vico Fallani et al., 2017). Additionally, it was recently shown that ECO is most effective at producing topologically representative brain networks, whose topology is robust to atlas type and size, for both binary and weighted networks (Luppi and Stamatakis, 2021). Here, the weight of the connection between pairs of nodes was quantified as the number of streamlines between them. For our validation analysis, we also constructed binary networks, by setting all non-zero edge weights to unity.

### 2.8 Self-similarity across scales: Fractal dimension from box covering algorithm

For all but the simplest real-world systems, fractal dimension cannot be computed analytically. Thus, a variety of so-called box-covering algorithms for complex networks (BCAN) have been developed as a means to approximate a network’s fractal dimension (Kim et al., 2007, Schneider et al., 2012, Song et al., 2007, Wei et al., 2013). These measures work by covering the network with the minimum possible number *N_B_* of “boxes” of a given size *l_B_.* For binary networks, a box is a collection of connected nodes whose distance (minimum number of edges that need to be traversed to move between them, also known as the shortest path) is less than the box size (Kim et al., 2007, Schneider et al., 2012, Song et al., 2007, Wei et al., 2013).

For a network of $N_{0}$ nodes, power-law fit between the box size *l_B_* and the minimum number *N_B_* of boxes of that size that are required to fully “tile” the network, is then used to estimate the network’s fractal dimension $d_{B}$ (Kim et al., 2007, Schneider et al., 2012, Song et al., 2007, Wei et al., 2013):

(2) $$N_{B}\left(l_{B}\right)=N_{0}{l_{B}}^{-d_{B}}$$

#### 2.8.1 Box-covering algorithm for weighted networks

Fractal dimension is influenced by connection weights (Wei et al., 2013). Since the strength of structural connectivity (number of white matter streamlines) differs across brain regions, here we rely on an improved box-covering algorithm that allows the information about connection weights to be taken into account (Wei et al., 2013), although we also validate our results using binary networks.

The key aspect of this box-covering algorithm for weighted networks (BCANw), is that values of the box size *l_B1_* …*l_Bn_* are not necessarily integers; rather, the various box sizes are obtained by progressively accumulating the minimum distance between nodes (shortest path), starting from the smallest individual shortest path, up to a maximum box size *l_Bn_* for which the network diameter (longest shortest path) is exceeded (Wei et al., 2013).

For each value of the box size *l_B_*, we can obtain a network composed of all the shortest paths *d_ij_* > *l_B_* from the original network; the number of boxes *N_B_* is then computed by applying a graph-coloring algorithm to this new network, such that *N_B_* is equal to the number of colors required to fully color the graph (Wei et al., 2013). Thereafter, fractal dimension is computed from power-law fit of number of boxes versus size of boxes, as per Equation (1) (log–log plots of box number vs box size for each participant are shown in Supplementary Figs. 1-4).

In box-covering approaches, the box size can be considered as the lens through which the network is being observed: a smaller size indicates that the system is being observed at higher resolution, and therefore at a smaller scale. Intuitively, in a network with fractal character the structure of boxes covering a given portion of the network should be preserved in the structure of larger boxes covering the entire network. In other words, if a system exhibits fractal character, then “zooming in” on its parts should reveal the same structure that characterises the whole. For this reason, fractal dimension constitutes a measure of similarity across scales.

### 2.9 Statistical analysis

Statistical significance of differences in fractal dimension based on diagnosis was assessed by conducting a three-way analysis of covariance (ANCOVA), testing for the effect of interest (diagnostic condition, with three levels: control, MCS and UWS) while controlling for DWI sequence type (12 vs 63 directions) and number of removed volumes due to motion corruption, as covariates of no interest. Upon finding the effect of interest to be statistically significant, we conducted post-hoc tests using three pairwise comparisons between the conditions (control vs. MCS, control vs. UWS, and MCS vs. UWS) while still controlling for DWI sequence type and number of removed volumes. We adopted the method of Benjamini and Hochberg (Benjamini and Hochberg, 1995) to control the false discovery rate across these three pairwise comparisons, at a two-sided alpha value of 0.05. The same covariates of no interest (DWI sequence type and number of removed volumes) were also included when comparing FMRI+ and FMRI- patients. To ensure the robustness of our results, we also carried out a validation analysis without including the covariates and using permutation-based testing to ensure robustness to outliers (two-sided between-subjects t-tests with 10,000 permutations); effect size was estimated using Cohen’s *d*.

## 3 Results

### 3.1 Reduced fractal dimension in patients with disorders of consciousness

**Fig. 2:**
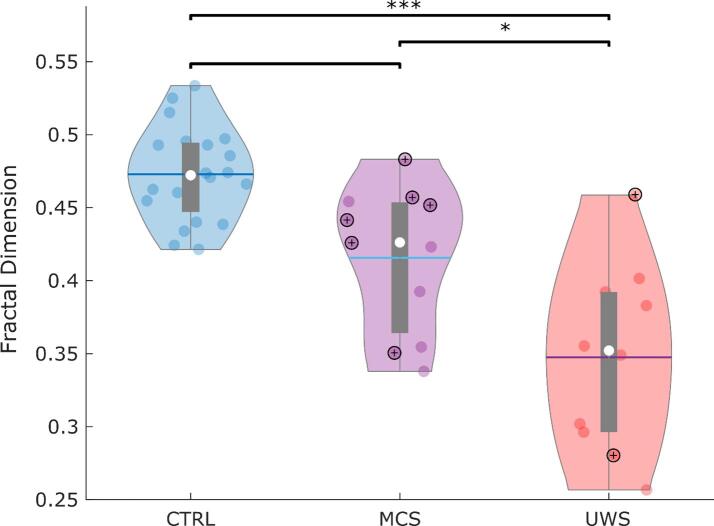
Reduced structural fractal dimension across disorders of consciousness. Violin plots indicate the distribution of weighted fractal dimension of structural brain networks for healthy controls (CTRL), minimally conscious patients (MCS), and patients diagnosed with unresponsive wakefulness syndrome (UWS). Circles with “+” signs indicate DOC patients who provided evidence of covert consciousness by performing mental imagery tasks in the scanner. White circle, median; blue center line, mean; box limits, upper and lower quartiles; whiskers, 1.5x interquartile range. * *p* < 0.05; *** *p* < 0.001, FDR-corrected across three pairwise comparisons. (For interpretation of the references to color in this figure legend, the reader is referred to the web version of this article.)

We compared weighted fractal dimension of structural brain networks across N = 20 healthy controls and N = 21 DOC patients belonging to different diagnostic categories (N = 11 MCS and N = 10 UWS). Analysis of covariance indicated a significant effect of condition (control, MCS, UWS) on weighted fractal dimension (FD): *F*(2,36) = 19.53, *p* < 0.001. Follow-up FDR-corrected pairwise tests indicated that UWS patients had significantly lower fractal dimension of structural brain networks than both healthy controls and MCS patients (Fig. 2 and Table 2). The difference between DOC patients with different aetiologies (traumatic vs anoxic injury) was not significant at the standard alpha value of 0.05, although this result should be interpreted with caution given the small group sizes (Supplementary Table 1).

**Table 2:** Statistical comparisons of weighted fractal dimension between pairs of conditions, using the Schaefer-1000 parcellation.

Contrast	Estimate	SE	tStat	EffSize	pVal
CTRL vs MCS	−0.035	0.019	−1.845	−0.331	0.076
CTRL vs UWS	−0.154	0.021	−7.315	−1.336	<0.001
MCS vs UWS	−0.068	0.027	−2.535	−0.553	0.021

### 3.2 Preserved fractal dimension in patients exhibiting functional MRI evidence of consciousness

Intriguingly, Fig. 2 reveals that out of 8 FMRI+ DOC patients, i.e., those who were able to demonstrate evidence of consciousness by responding to mental imagery tasks, 6 have a structural fractal dimension in the range of healthy controls.

We therefore investigated whether ability to provide evidence of consciousness by successfully performing mental imagery tasks in the MRI scanner (Monti et al., 2010, Owen et al., 2006) is associated with preserved fractal dimension of the structural connectome. Indeed, we found that the structural brain networks of FMRI+ patients had significantly higher weighted fractal dimension than those of FMRI- patients (Table 3), who had failed to provide evidence of consciousness in terms of responsiveness to mental imagery tasks (note that classification as FMRI+ or FMRI- is independent of overt motor responses, i.e. the criterion on which patients’ diagnoses are based).

**Table 3:** Statistical comparisons of weighted fractal dimension between FMRI+ and FMRI- DOC patients, using the Schaefer-1000 parcellation.

Contrast	Estimate	SE	tStat	EffSize	pVal
FMRI+ vs FMRI-	−0.064	0.026	−2.463	−0.537	0.025

### 3.3 Validation of results

To ensure the robustness of our results, we repeated our main analysis with permutation testing, without including the covariates of no interest. The results replicated our main analysis, including the significant difference between FMRI+ and FMRI- patients, but also identified a significant difference (FDR-corrected) between healthy controls and MCS patients (Supplementary Figure 5 and Supplementary Table 2), which had become narrowly non-significant in our main analysis, after accounting for the covariates.

To further ensure that our results were not critically dependent on our choice of parcellation type, parcellation side, or edge definition, we varied each of these aspects in turn: we replicated our analyses with the largest scale of the Lausanne parcellation (with 1000 cortical nodes) (Cammoun et al., 2012); with the 400-ROI version of the Schaefer parcellation (Schaefer et al., 2018); with binary rather than weighted edges; and with edges derived from probabilistic tractography performed in native space (Supplementary Figures 6–9 and Supplementary Tables 3-6); all these further analyses were controlled for the same covariates of no interest (DWI acquisition type and number of motion-corrupted scans).

Compared with our main analysis, alternative analyses using 400 nodes or binary edges both found additional significant differences (FDR-corrected) between healthy controls and MCS patients; conversely, fewer statistically significant differences were observed with the Lausanne parcellation and with alternative tractography: in both cases, the difference between MCS and UWS patients failed to reach statistical significance. Nevertheless, all analyses consistently revealed significant reductions in the fractal dimension of structural networks in UWS patients compared with healthy controls, demonstrating the robustness of this effect. Crucially, across our validation analyses the majority of FMRI+ patients consistently exhibited structural fractal dimension in the range of healthy controls, significantly higher than FMRI- patients.

## 4 Discussion

Here, we investigated whether the fractal dimension of structural brain networks is reduced in patients suffering from disorders of consciousness due to severe brain injury. Supporting previous findings pertaining to functional brain networks (Varley et al., 2020b), our results indicate that impaired consciousness is reflected in a reduction in the self-similarity across scales (fractal dimension) of the brain, when viewed as a network of anatomical connections. Specifically, the structural connectomes of patients with more severe disorders of consciousness (UWS as opposed to MCS) are less self-similar across scales. The observation of reduced structural fractal dimension in patients suffering from disorders of consciousness is robust to how nodes and edges in the networks are defined. Some of our alternative approaches also identified subtler differences between healthy controls and MCS patients, and between MCS and UWS patients (although replication in a larger sample will be required). We note that in order to avoid reductions in our limited sample size, we included in this analysis DTI data from two different DTI acquisitions. However, we explicitly controlled for this potential confound in our statistical models. Additionally, we are reassured by the fact that both acquisition cohorts comprised MCS and UWS patients, with both traumatic and anoxic brain injury, and including both FMRI+ and FMRI- patients.

Though widely used to clinically assess a patient’s level of consciousness, behavioural responsiveness to commands relies on patients’ ability to carry out the required motor actions. As a result, clinical diagnosis of DOC patients is fraught with difficulties, and up to 40% of misdiagnoses have been reported (Naci et al., 2017). By bypassing the need for motor responses, mental imagery tasks in the scanner have proved efficacious to detect residual, “covert” consciousness despite a patient’s inability to overtly execute motor responses (Monti et al., 2010, Owen et al., 2006).

It has been argued that fractal organisation is crucial in balancing the brain’s opposing needs for integration and differentiation (Gallos et al., 2012b, Gallos et al., 2012, Ruiz de Miras et al., 2019) – two key requirements for consciousness, according to leading theoretical work (Balduzzi and Tononi, 2008, Tononi et al., 1994). Crucially, fractal dimension of the structural connectome was associated not only with clinical diagnosis (based on overt behavioural responsiveness), but also with patients’ ability to provide evidence of consciousness by performing mental imagery tasks in the MRI scanner (Monti et al., 2010, Owen et al., 2006).

Intriguingly, these results complement recent evidence from functional MRI in the same patients (Luppi et al., 2020b), which showed that the brain dynamics of FMRI+ patients are not significantly different from those of healthy controls – whereas both healthy controls and FMRI+ patients are significantly different from FMRI-, whose brain dynamics resemble those of anaesthetised volunteers. The technique used in that study (Luppi et al., 2020b), known as “connectome harmonic decomposition”, relates brain dynamics to the underlying connectome (Atasoy et al., 2016). Although a group-average healthy connectome was used (Luppi et al., 2020b), future connectome harmonic research may seek to identify whether the difference in fractal character of patients’ individual connectomes may contribute to explain the corresponding differences in brain dynamics.

Thus, the present findings support the notion that severe brain injury may induce loss of consciousness due to compromised information transmission and processing capabilities of the human brain, as is also suggested by work using functional approaches (Luppi et al., 2019, Varley et al., 2020b). Importantly, fractal dimension of the structural connectome is based entirely on brain anatomy rather than function, and therefore measuring the structural fractal dimension does not impose any linguistic, volitional or cognitive demands on patients – in fact, it does not even depend on patients’ levels of arousal, which is crucial for functional data and assessment of responsiveness. Additionally, structural brain networks may be expected to be less variable over time than functional ones, which are known to be inherently dynamic and variable from moment to moment (Allen et al., 2014, Demertzi et al., 2019, Luppi et al., 2019, Zamani Esfahlani et al., 2020); indeed structural networks may exhibit higher reproducibility over time than functional ones (Lawrence et al., 2018), which may provide additional advantages as a prognostic tool. Future longitudinal studies may investigate whether recovery of consciousness is associated with a corresponding recovery of fractal dimension of the structural connectome, and whether the latter is predictive of the former within individual patients.

Although the small sample size of the present study warrants caution and calls for replication in larger cohorts, we tentatively propose that this neuroanatomical measure may represent a useful addition to behavioural assessments in the clinic: DOC patients whose structural fractal dimension is comparable to that of a healthy brain, may be especially promising candidates for more in-depth examination, such as by means of mental imagery tasks in the scanner (Monti et al., 2010, Owen et al., 2006, Craig et al., 2021), task-free paradigms (Naci et al., 2017), or other indices of residual consciousness such as the Perturbational Complexity Index (Casali et al., 2013). Of course, some FMRI- patients exhibited preserved fractal dimension, and not all FMRI+ patients had high levels of structural fractal dimension. Likewise, some FMRI+ patients were behaviourally characterised as unresponsive (UWS diagnosis), as were some patients with relatively preserved fractal dimension. Such variability is not surprising: DOCs are highly heterogeneous conditions, not only in terms of severity, but also varying in the cause, location and extent of brain damage.

Therefore, it is important to emphasise that here we do not advocate for assessment of structural fractal dimension as being an alternative to either clinical assessment or fMRI task-based assessment of covert consciousness: rather, we view these various measures as providing complementary insights. Likewise, here we do not claim that high fractal dimension of the structural connectome is either sufficient nor necessary for the presence of consciousness – although the present findings do suggest that an association exists between these aspects, in line with theoretical proposals. Future work employing personalised medicine approaches and computational modelling (Cofré et al., 2020, Kringelbach and Deco, 2020, Luppi et al., 2021) may provide additional insights into the origin and clinical significance of this association, by identifying patient-specific sources of reduced structural fractal dimension.

### 4.1 Conclusion

Overall, we demonstrate that disorders of consciousness arising from severe brain injury correspond to reduced self-similarity across scales in the network organisation or the brain’s anatomical connections. However, we also show that structural fractal dimension is preserved in patients who are able to provide evidence of consciousness by performing mental imagery tasks in the scanner. It is our hope that this measure may prove to have prognostic value in the clinic, complementing measures that rely on patients’ ability to overtly or covertly respond to commands.

## CRediT authorship contribution statement

**Andrea I. Luppi:** Conceptualization, Formal analysis, Investigation, Methodology, Project administration, Software, Writing - original draft. **Michael M. Craig:** Data curation, Formal analysis, Investigation, Software. **Peter Coppola:** Investigation, Writing - review & editing. **Alexander R.D. Peattie:** Investigation, Writing - review & editing. **Paola Finoia:** Data curation. **Guy B. Williams:** Data curation, Funding acquisition. **Judith Allanson:** Data curation, Funding acquisition, Writing - review & editing. **John D. Pickard:** Data curation, Funding acquisition. **David K. Menon:** Data curation, Funding acquisition, Methodology, Project administration, Supervision, Writing - review & editing. **Emmanuel A. Stamatakis:** Conceptualization, Data curation, Funding acquisition, Methodology, Supervision, Writing - review & editing.

## Declaration of Competing Interest

The authors declare that they have no known competing financial interests or personal relationships that could have appeared to influence the work reported in this paper.

## References

[b0005] Allen E.A., Damaraju E., Plis S.M., Erhardt E.B., Eichele T., Calhoun V.D. (2014). Tracking whole-brain connectivity dynamics in the resting state. Cereb. Cortex.

[b0010] Atasoy S., Donnelly I., Pearson J. (2016). Human brain networks function in connectome-specific harmonic waves. Nat. Commun..

[b0015] Balduzzi D., Tononi G. (2008). Integrated information in discrete dynamical systems: Motivation and theoretical framework. PLoS Comput. Biol..

[b0020] Benjamini Y., Hochberg Y. (1995). Controlling the False Discovery Rate: A Practical and Powerful Approach to Multiple Testing. J. R. Stat. Soc. Ser. B.

[b0025] Bettinardi R.G., Deco G., Karlaftis V.M., Van Hartevelt T.J., Fernandes H.M., Kourtzi Z., Kringelbach M.L., Zamora-López G. (2017). How structure sculpts function: Unveiling the contribution of anatomical connectivity to the brain’s spontaneous correlation structure. Chaos.

[b0030] Bornas X., Tortella-Feliu M., Balle M., Llabrés J. (2013). Self-focused cognitive emotion regulation style as associated with widespread diminished EEG fractal dimension. Int. J. Psychol..

[b0035] Bruno M.-A., Vanhaudenhuyse A., Thibaut A., Moonen G., Laureys S. (2011). From unresponsive wakefulness to minimally conscious PLUS and functional locked-in syndromes: recent advances in our understanding of disorders of consciousness. J. Neurol..

[b0040] Cammoun L., Gigandet X., Meskaldji D., Thiran J.P., Sporns O., Do K.Q., Maeder P., Meuli R., Hagmann P. (2012). Mapping the human connectome at multiple scales with diffusion spectrum MRI. J. Neurosci. Methods.

[b0045] Carhart-Harris R.L. (2018). The entropic brain - revisited. Neuropharmacology.

[b0050] Carhart-Harris R.L., Leech R., Hellyer P.J., Shanahan M., Feilding A., Tagliazucchi E., Chialvo D.R., Nutt D. (2014). The entropic brain: a theory of conscious states informed by neuroimaging research with psychedelic drugs. Front. Hum. Neurosci..

[b0055] Casali A.G., Gosseries O., Rosanova M., Boly M., Sarasso S., Casali K.R., Casarotto S., Bruno M.-A., Laureys S., Tononi G., Massimini M. (2013). A theoretically based index of consciousness independent of sensory processing and behavior. Sci. Transl. Med..

[b0060] Cavaliere C., Aiello M., Perri C.D., Fernandez-Espejo D., Owen A.M., Soddu A. (2015). Diffusion tensor imaging and white matter abnormalities in patients with disorders of consciousness. Front. Hum. Neurosci..

[b0065] Chen Y. (2020). Equivalent relation between normalized spatial entropy and fractal dimension. Physica A.

[bib342] Cofré R., Herzog R., Mediano P.A.M., Piccinini J., Rosas F.E., Perl Y.S., Tagliazucchi E. (2020). Whole-brain models to explore altered states of consciousness from the bottom up. Brain Sci..

[b0070] Correia M.M., Carpenter T.A., Williams G.B. (2009). Looking for the optimal DTI acquisition scheme given a maximum scan time: are more b-values a waste of time?. Magn. Reson. Imaging.

[bib341] Craig, M.M., Pappas, I., Allanson, J., Finoia, P., Williams, G., Pickard, J.D., Menon, D.K., Stamatakis, E.A., 2021. Resting-state based prediction of task-related activation in patients with disorders of consciousness. bioRxiv 2021.03.27.436534. 10.1101/2021.03.27.436534.

[b0075] De Vico Fallani F., Latora V., Chavez M. (2017). A Topological Criterion for Filtering Information in Complex Brain Networks. PLoS Comput. Biol..

[b0080] Deco G., Jirsa V.K. (2012). Ongoing cortical activity at rest: Criticality, multistability, and ghost attractors. J. Neurosci..

[b0085] Demertzi A., Martial C., Demertzi A., Tagliazucchi E., Dehaene S., Deco G., Barttfeld P., Raimondo F., Martial C., Fernández-Espejo D., Rohaut B., Voss H.U., Schiff N.D., Owen A.M., Laureys S., Naccache L., Sitt J.D. (2019). Human consciousness is supported by dynamic complex patterns of brain signal coordination. Sci. Adv..

[b0090] Fernández-Espejo D., Bekinschtein T., Monti M.M., Pickard J.D., Junque C., Coleman M.R., Owen A.M. (2011). Diffusion weighted imaging distinguishes the vegetative state from the minimally conscious state. Neuroimage.

[b0095] Fernández-Espejo, D., Norton, L., Owen, A.M., 2014. The clinical utility of fMRI for identifying covert awareness in the vegetative state: A comparison of sensitivity between 3T and 1.5T. PLoS One 9. https://doi.org/10.1371/journal.pone.0095082. 10.1371/journal.pone.0095082 PMC3986373 24733575

[b0100] Fernández-Espejo D., Soddu A., Cruse D., Palacios E.M., Junque C., Vanhaudenhuyse A., Rivas E., Newcombe V., Menon D.K., Pickard J.D., Laureys S., Owen A.M. (2012). A role for the default mode network in the bases of disorders of consciousness. Ann. Neurol..

[b0105] Gallos L.K., Makse H.A., Sigman M. (2012). A small world of weak ties provides optimal global integration of self-similar modules in functional brain networks. Proc. Nati. Acad. Sci. USA.

[b0110] Gallos, L.K., Sigman, M., Makse, H.A., 2012b. The conundrum of functional brain networks: Small-world efficiency or fractal modularity. Front. Physiol. 3 MAY. https://doi.org/10.3389/fphys.2012.00123. 10.3389/fphys.2012.00123 PMC3345943 22586406

[b0115] Gu S., Betzel R.F., Mattar M.G., Cieslak M., Delio P.R., Grafton S.T., Pasqualetti F., Bassett D.S. (2017). Optimal trajectories of brain state transitions. Neuroimage.

[b0120] Gu S., Pasqualetti F., Cieslak M., Telesford Q.K., Yu A.B., Kahn A.E., Medaglia J.D., Vettel J.M., Miller M.B., Grafton S.T., Bassett D.S. (2015). Controllability of structural brain networks. Nat. Commun..

[b0125] Hagmann P., Cammoun L., Gigandet X., Meuli R., Honey C.J. (2008). Mapping the structural core of human cerebral cortex. PLoS Biol.

[b0130] Im K., Lee J.-M., Yoon U., Shin Y.-W., Hong S.B., Kim I.Y., Kwon J.S., Kim S.I. (2006). Fractal dimension in human cortical surface: Multiple regression analysis with cortical thickness, sulcal depth, and folding area. Hum. Brain Mapp..

[b0135] Kim J.S., Goh K.-I., Kahng B., Kim D. (2007). A box-covering algorithm for fractal scaling in scale-free networks. Chaos.

[b0140] King R.D., George A.T., Jeon T., Hynan L.S., Youn T.S., Kennedy D.N., Dickerson B. (2009). Characterization of atrophic changes in the cerebral cortex using fractal dimensional analysis. Brain Imag. Behav..

[b0145] Klonowski, W., Olejarczyk, E., Stępień, R., 2005. Sleep-EEG Analysis Using Higuchi ’ s Fractal Dimension.

[bib343] Kringelbach M.L., Deco G. (2020). Brain States and Transitions: Insights from Computational Neuroscience. Cell Rep..

[b0150] Kuceyeski A., Shah S., Dyke J.P., Bickel S., Abdelnour F., Schiff N.D., Voss H.U., Raj A. (2016). The application of a mathematical model linking structural and functional connectomes in severe brain injury. NeuroImage Clin..

[b0155] Lant N.D., Gonzalez-Lara L.E., Owen A.M., Fernández-Espejo D. (2016). Relationship between the anterior forebrain mesocircuit and the default mode network in the structural bases of disorders of consciousness. NeuroImage Clin..

[b0160] Lawrence A.J., Tozer D.J., Stamatakis E.A., Markus H.S. (2018). A comparison of functional and tractography based networks in cerebral small vessel disease. NeuroImage Clin..

[b0165] Le Bihan D., Johansen-Berg H. (2012). Diffusion MRI at 25: Exploring brain tissue structure and function. Neuroimage.

[b0170] Leemans A., Jones D.K. (2009). The B-matrix must be rotated when correcting for subject motion in DTI data. Magn. Reson. Med..

[b0175] Luppi A.I., Craig M.M., Pappas I., Finoia P., Williams G.B., Allanson J., Pickard J.D., Owen A.M., Naci L., Menon D.K., Stamatakis E.A. (2019). Consciousness-specific dynamic interactions of brain integration and functional diversity. Nat. Commun..

[b0180] Luppi, A.I., Mediano, P.A., Rosas, F.E., Allanson, J., Carhart-Harris, R.L., Williams, G.B., Craig, M.M., Finoia, P., Owen, A.M., Naci, L., Menon, D.K., Bor, D., Stamatakis, E.A., 2020a. A Synergistic Workspace for Human Consciousness Revealed by Integrated Information Decomposition. bioRxiv 2020.11.25.398081. https://doi.org/10.1101/2020.11.25.398081. 10.7554/eLife.88173 PMC11257694 39022924

[bib344] Luppi A.I., Mediano P.A., Rosas F.E., Allanson J., Williams G.B., Craig M.M., Finoia P., Peattie A.R., Coppola P., Owen A., Naci L., Menon D.K., Bor D. (2021). Paths to Oblivion: Common Neural Mechanisms of Anaesthesia and Disorders of Consciousness. biorXiv.

[b0185] Luppi A.I., Stamatakis E.A. (2021). Combining network topology and information theory to construct representative brain networks. Netw. Neurosci..

[b0190] Luppi, A.I., Vohryzek, Jakub, Kringelbach, M.L., Mediano, P.A., Craig, M.M., Adapa, R., Carhart-Harris, R.L., Roseman, L., Pappas, I., Finoia, P., Williams, G.B., Allanosn, J., Pickard, J.D., Menon, D.K., Atasoy, S., Stamatakis, E.A., 2020b. Connectome Harmonic Decomposition of Human Brain Dynamics Reveals a Landscape of Consciousness. bioRxiv. https://doi.org/10.1101/2020.08.10.244459.

[b0195] A.A. MacDonald L. Naci P.A. MacDonald A.M. Owen Anesthesia and neuroimaging: Investigating the neural correlates of unconsciousness 2015 Sci Trends Cogn 10.1016/j.tics.2014.12.005. 10.1016/j.tics.2014.12.005 25592916

[b0200] Medaglia, J.D., Gu, S., Pasqualetti, F., Ashare, R.L., Lerman, C., Kable, J., Bassett, D.S., 2016. Cognitive Control in the Controllable Connectome. arXiv.

[b0205] Monti M.M., Vanhaudenhuyse A., Coleman M.R., Boly M., Pickard J.D., Tshibanda L., Owen A.M., Laureys S. (2010). Willful modulation of brain activity in disorders of consciousness. N. Engl. J. Med..

[b0210] Mustafa N., Ahearn T.S., Waiter G.D., Murray A.D., Whalley L.J., Staff R.T. (2012). Brain structural complexity and life course cognitive change. Neuroimage.

[b0215] Naci L., Sinai L., Owen A.M. (2017). Detecting and interpreting conscious experiences in behaviorally non-responsive patients. Neuroimage.

[b0220] Newcombe V.F.J., Williams G.B., Scoffings D., Cross J., Carpenter T.A., Pickard J.D., Menon D.K. (2010). Aetiological differences in neuroanatomy of the vegetative state: Insights from diffusion tensor imaging and functional implications. J. Neurol. Neurosurg. Psychiatry.

[b0225] Owen A.M., Coleman M.R., Boly M., Davis M.H., Laureys S., Pickard J.D. (2006). Detecting awareness in the vegetative state. Science (80-.)..

[b0230] Ruiz de Miras J., Soler F., Iglesias-Parro S., Ibáñez-Molina A.J., Casali A.G., Laureys S., Massimini M., Esteban F.J., Navas J., Langa J.A. (2019). Fractal dimension analysis of states of consciousness and unconsciousness using transcranial magnetic stimulation. Comput. Methods Programs Biomed..

[b0235] Schaefer A., Kong R., Gordon E.M., Laumann T.O., Zuo X.-N., Holmes A.J., Eickhoff S.B., Yeo B.T.T. (2018). Local-Global Parcellation of the Human Cerebral Cortex from Intrinsic Functional Connectivity MRI. Cereb. Cortex.

[b0240] Schneider C.M., Kesselring T.A., Andrade J.S., Herrmann H.J. (2012). Box-covering algorithm for fractal dimension of complex networks. Phys. Rev. E - Stat. Nonlinear, Soft Matter Phys..

[b0245] Song C., Gallos L.K., Havlin S., Makse H.A. (2007). How to calculate the fractal dimension of a complex network: The box covering algorithm. J. Stat. Mech. Theory Exp..

[b0250] Spasic S., Kesic S., Kalauzi A., Saponjic J. (2011). Different anesthesia in rat induces distinct inter-structure brain dynamic detected by Higuchi fractal dimension. Fractals.

[b0255] Sporns, O., Tononi, G., Kötter, R., 2005. The human connectome: A structural description of the human brain. PLoS Comput. Biol. https://doi.org/10.1371/journal.pcbi.0010042. 10.1371/journal.pcbi.0010042 PMC1239902 16201007

[b0260] Tae H.H., Yoon U., Kyung J.L., Yong W.S., Lee J.M., In Y.K., Kyoo S.H., Kim S.I., Jun S.K. (2005). Fractal dimension of cerebral cortical surface in schizophrenia and obsessive-compulsive disorder. Neurosci. Lett..

[b0265] Tan X., Zhou Z., Gao J., Meng F., Yu Y., Zhang J., He F., Wei R., Wang J., Peng G., Zhang X., Pan G., Luo B. (2019). Structural connectome alterations in patients with disorders of consciousness revealed by 7-tesla magnetic resonance imaging. NeuroImage Clin..

[b0270] Tononi G., Sporns O., Edelman G.M. (1994). A measure for brain complexity: relating functional segregation and integration in the nervous system. Proc. Natl. Acad. Sci..

[b0275] Tournier J.D., Smith R., Raffelt D., Tabbara R., Dhollander T., Pietsch M., Christiaens D., Jeurissen B., Yeh C.H., Connelly A. (2019). MRtrix3: A fast, flexible and open software framework for medical image processing and visualisation. Neuroimage.

[b0280] Varley T.F., Carhart-Harris R., Roseman L., Menon D.K., Stamatakis E.A. (2020). Serotonergic psychedelics LSD & psilocybin increase the fractal dimension of cortical brain activity in spatial and temporal domains. Neuroimage.

[b0285] Varley T.F., Craig M.M., Adapa R., Finoia P., Williams G., Allanson J., Pickard J., Menon D.K., Stamatakis E.A. (2020). Fractal dimension of cortical functional connectivity networks & severity of disorders of consciousness. PLoS One.

[b0290] Varley T.F., Luppi A.I., Pappas I., Naci L., Adapa R., Owen A.M., Menon D.K., Stamatakis E.A. (2020). Consciousness & Brain Functional Complexity in Propofol Anaesthesia. Sci. Rep..

[b0295] Veraart J., Novikov D.S., Christiaens D., Ades-Aron B., Sijbers J., Fieremans E. (2016). Denoising of diffusion MRI using random matrix theory. Neuroimage.

[b0300] Wang L., Yang Y., Chen S., Ge M., He J., Yang Z., Lin P., Wu X. (2018). White matter integrity correlates with residual consciousness in patients with severe brain injury. Brain Imaging Behav..

[b0305] Wannez S., Gosseries O., Azzolini D., Martial C., Cassol H., Aubinet C., Annen J., Martens G., Bodart O., Heine L., Charland-Verville V., Thibaut A., Chatelle C., Vanhaudenhuyse A., Demertzi A., Schnakers C., Donneau A.-F., Laureys S. (2018). Prevalence of coma-recovery scale-revised signs of consciousness in patients in minimally conscious state. Neuropsychol. Rehabil..

[b0310] Wei D.J., Liu Q., Zhang H.X., Hu Y., Deng Y., Mahadevan S. (2013). Box-covering algorithm for fractal dimension of weighted networks. Sci. Rep..

[b0315] Weng L., Xie Q., Zhao L., Zhang R., Ma Q., Wang J., Jiang W., He Y., Chen Y., Li C., Ni X., Xu Q., Yu R., Huang R. (2017). Abnormal structural connectivity between the basal ganglia, thalamus, and frontal cortex in patients with disorders of consciousness. Cortex.

[b0320] Wu X., Zhang J., Cui Z., Tang W., Shao C., Hu J., Zhu J., Zhao Y., Lu L., Chen G., Northoff G., Gong G., Mao Y., He Y. (2018). White Matter Deficits Underlying the Impaired Consciousness Level in Patients with Disorders of Consciousness. Neurosci. Bull..

[b0325] Yeh F.-C., Verstynen T.D., Wang Y., Fernández-Miranda J.C., Tseng W.-Y. (2013). Deterministic Diffusion Fiber Tracking Improved by Quantitative Anisotropy. PLoS One.

[b0330] Yeh F.-C., Wedeen V.J., Tseng W.-Y.-I. (2011). Estimation of fiber orientation and spin density distribution by diffusion deconvolution. Neuroimage.

[b0335] Zamani Esfahlani F., Jo Y., Faskowitz J., Byrge L., Kennedy D.P., Sporns O., Betzel R.F. (2020). High-amplitude cofluctuations in cortical activity drive functional connectivity. Proc. Natl. Acad. Sci. USA.

[b0340] Zheng Z.S., Reggente N., Lutkenhoff E., Owen A.M., Monti M.M. (2017). Disentangling disorders of consciousness: Insights from diffusion tensor imaging and machine learning. Hum. Brain Mapp..

