# Supplementary material for: Preserved fractal character of structural brain networks is associated with covert consciousness after severe brain injury
Source: Neuroimage Clin. 2021 Apr 21;30:102682. doi: 10.1016/j.nicl.2021.102682 (PMC8102619; doi:10.1016/j.nicl.2021.102682)
Supplement: Supplementary data 1 [file mmc1.docx]

# Supplementary Information for

Preserved fractal character of structural brain networks is associated with covert consciousness after severe brain injury

Andrea I. Luppi^a,b^*, Michael M. Craig^a,b^, Peter Coppola^a,b^, Alexander R.D. Peattie^a,b^, Paola Finoia^a,c^, Guy B. Williams^b,d^, Judith Allanson^b,e^, John D. Pickard^b,c,d^, David K. Menon^a,d^, Emmanuel A. Stamatakis^a,b^

^a^Division of Anaesthesia, School of Clinical Medicine, University of Cambridge, Addenbrooke's Hospital, Hills Rd, CB2 0SP, Cambridge, United Kingdom

^b^Department of Clinical Neurosciences, School of Clinical Medicine, University of Cambridge, Addenbrooke's Hospital, Hills Rd, CB2 0SP, Cambridge, United Kingdom

^c^Division of Neurosurgery, School of Clinical Medicine, University of Cambridge, Addenbrooke's Hospital, Hills Rd, CB2 0SP, Cambridge, United Kingdom

^d^Wolfson Brain Imaging Centre, University of Cambridge, Cambridge Biomedical Campus (Box 65, Cambridge CB2 0QQ, United Kingdom

^e^Department of Neurosciences, Cambridge University Hospitals NHS Foundation, Addenbrooke's Hospital, Hills Rd, CB2 0SP, Cambridge, United Kingdom

*Corresponding author: email [al857@cam.ac.uk](mailto:al857@cam.ac.uk)

## Supplementary Figures


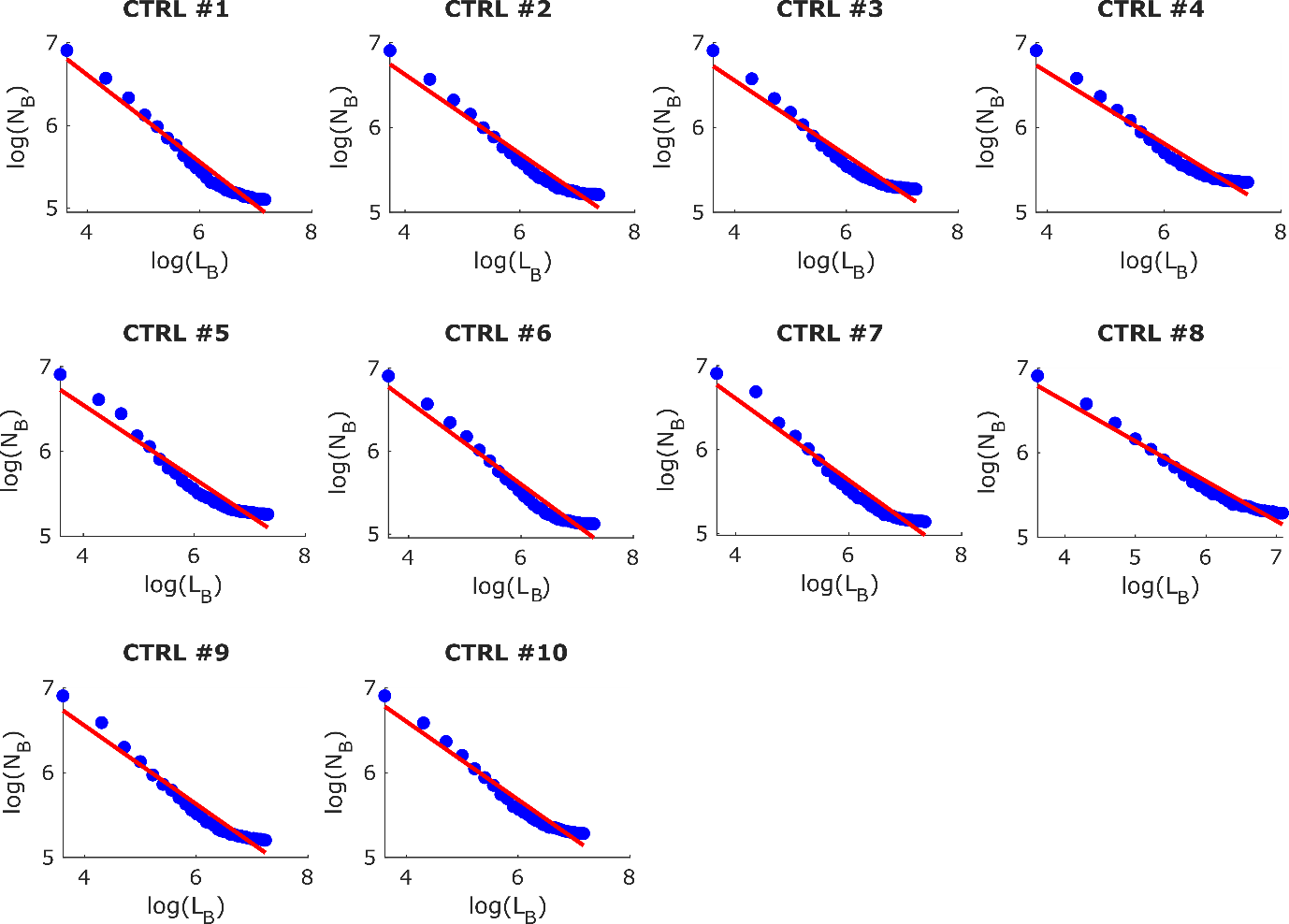


**Supplementary Figure 1. Log-log plots showing the box size *L_B_* and the minimum number *N_B_* of boxes of that size that are required to fully “tile” the network, for healthy controls #1-10.**


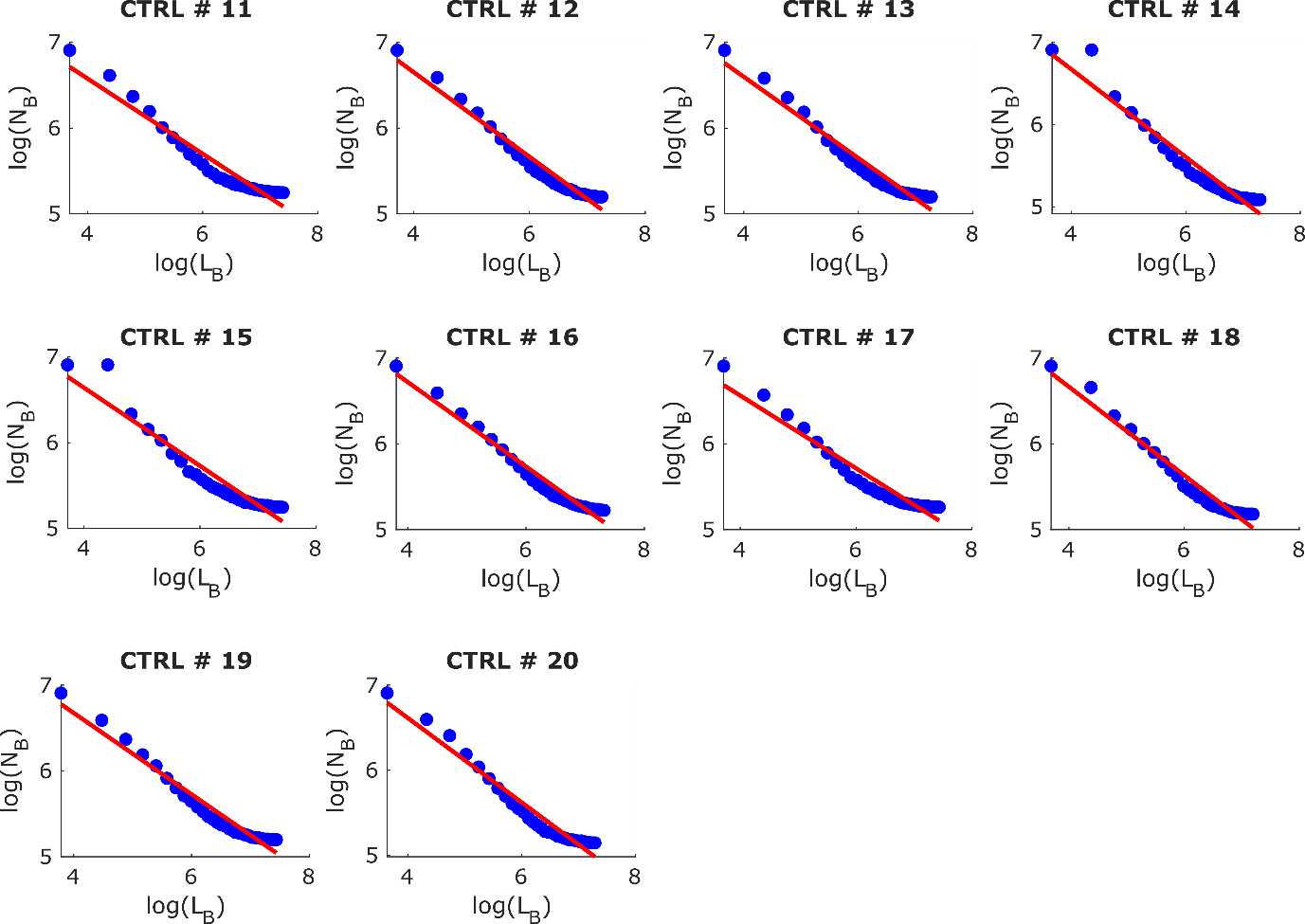


**Supplementary Figure 2. Log-log plots showing the box size *L_B_* and the minimum number *N_B_* of boxes of that size that are required to fully “tile” the network, for healthy controls #11-20.**


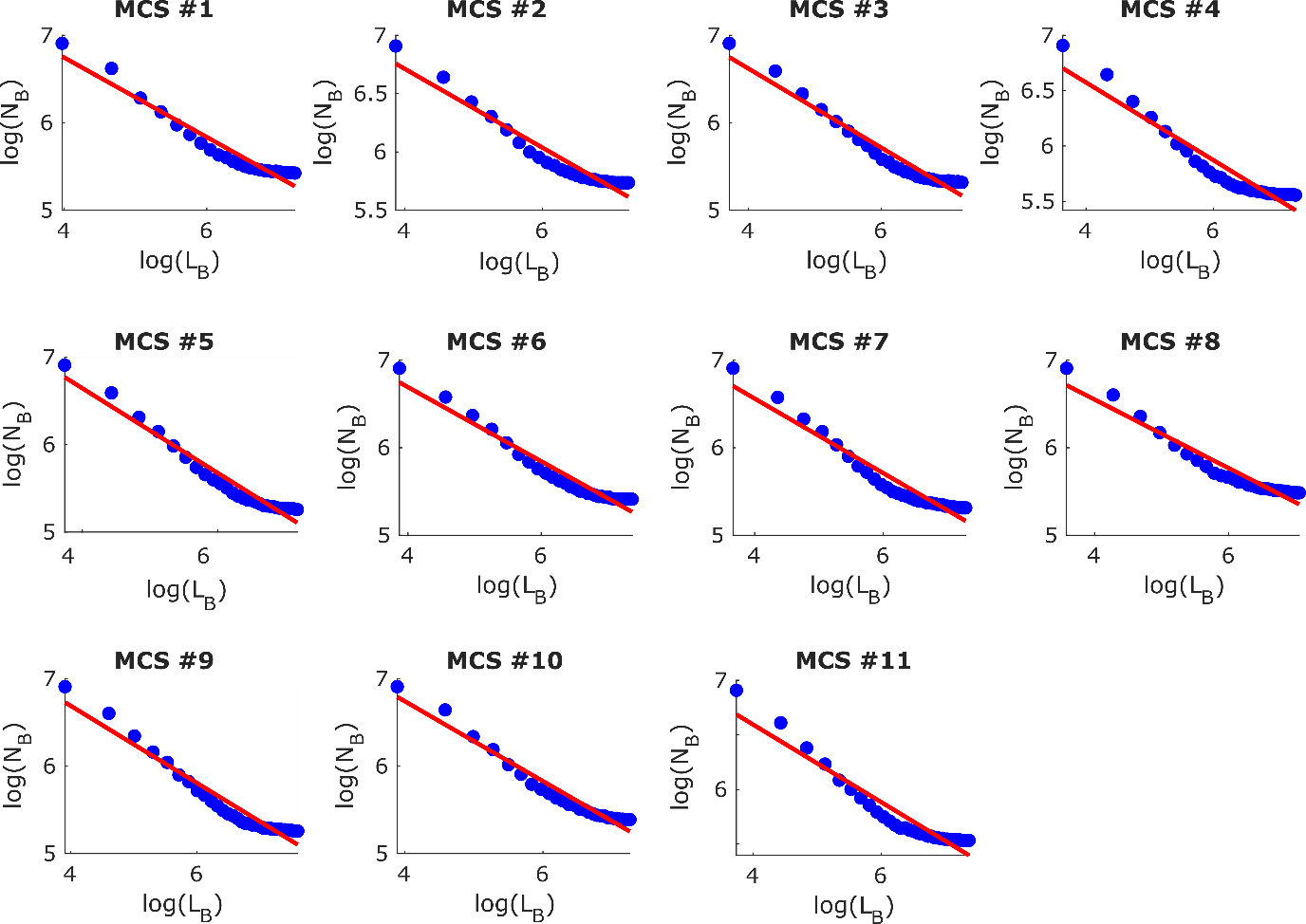


**Supplementary Figure 3. Log-log plots showing the box size *L_B_* and the minimum number *N_B_* of boxes of that size that are required to fully “tile” the network, for MCS patients**


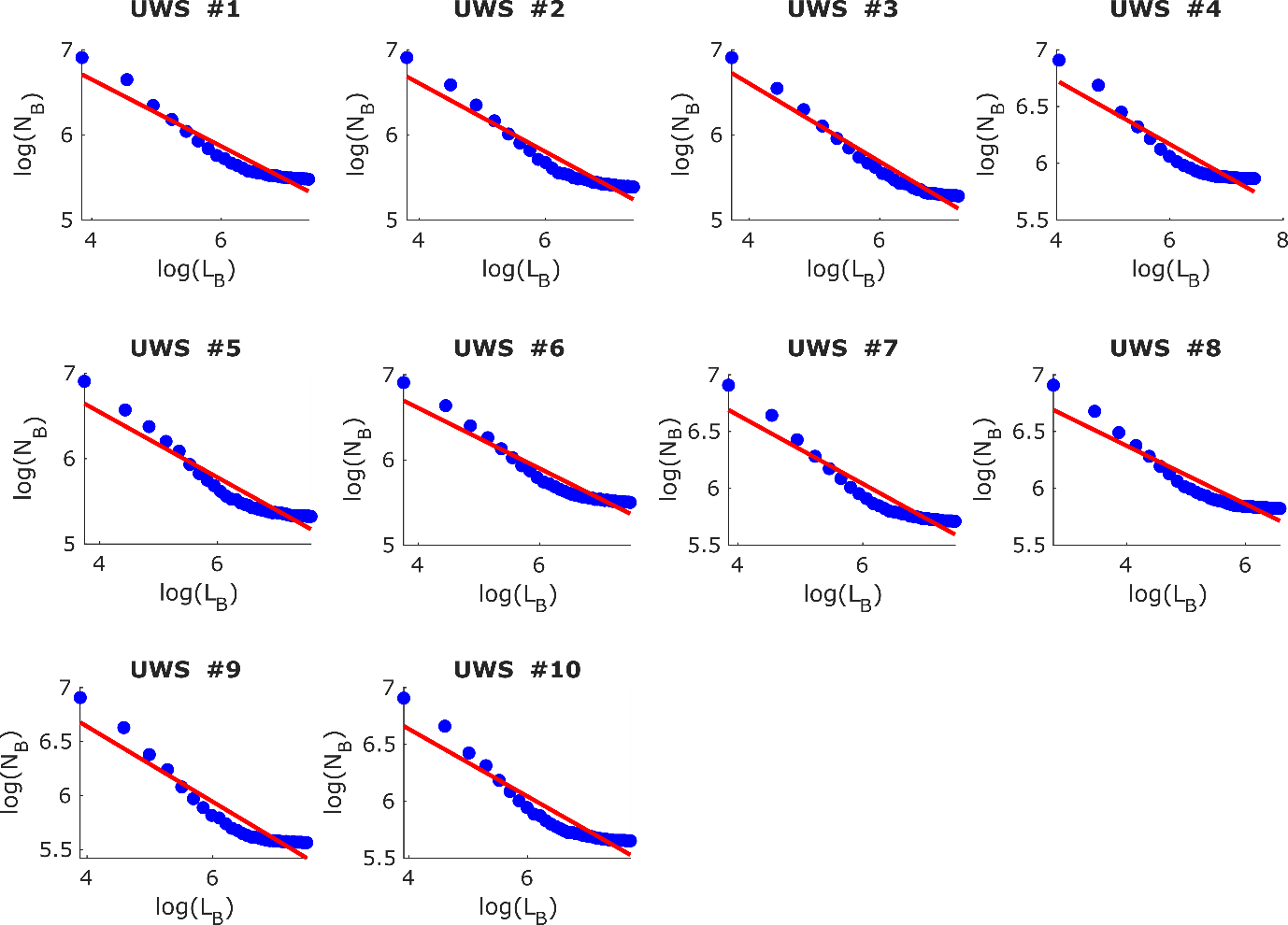


**Supplementary Figure 4. Log-log plots showing the box size *L_B_* and the minimum number *N_B_* of boxes of that size that are required to fully “tile” the network, for UWS patients.**


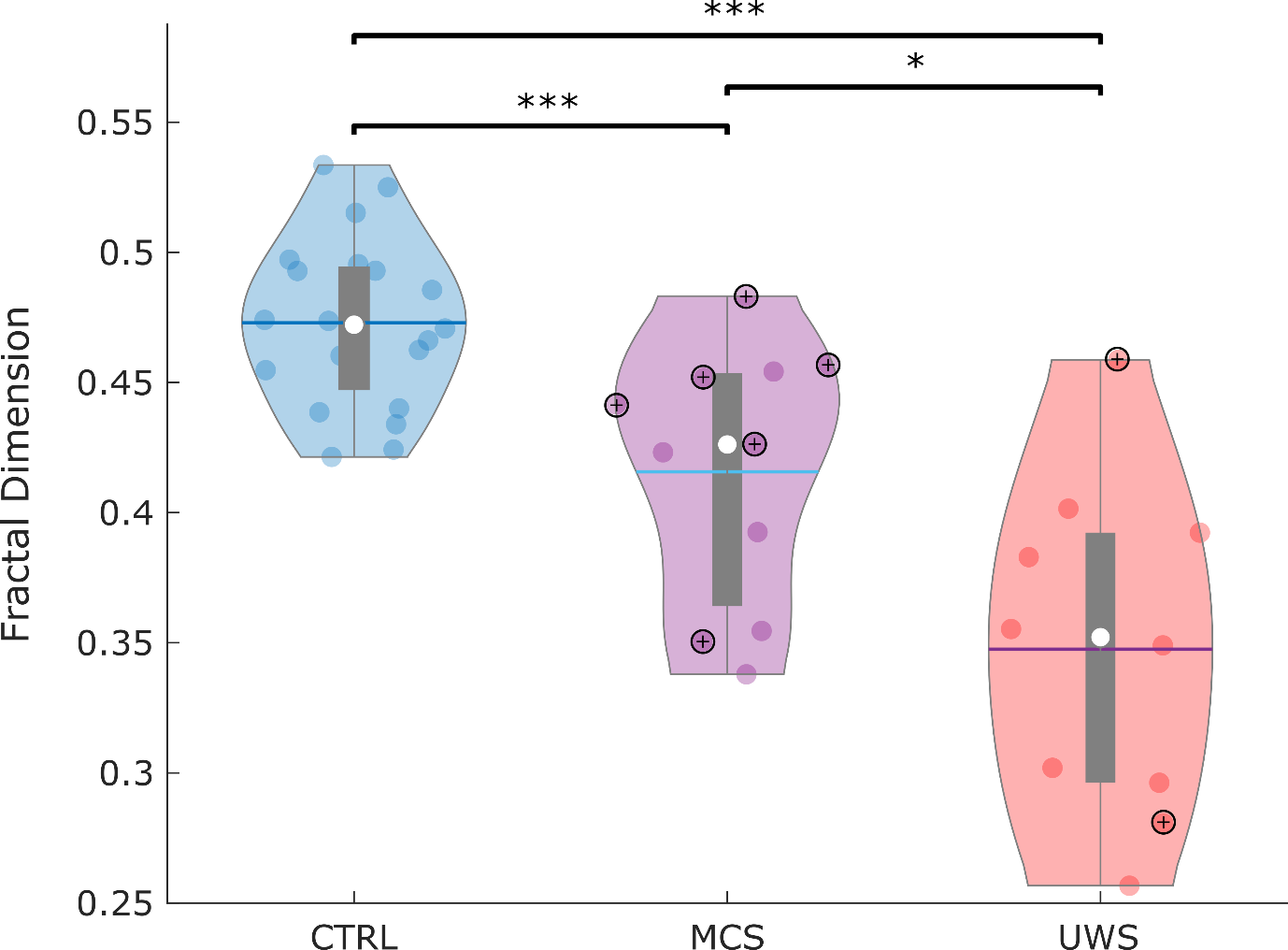


**Supplementary Figure 5. Reduced structural fractal dimension across disorders of consciousness, without including covariates.** Violin plots indicate the distribution of weighted fractal dimension of structural brain networks for healthy controls (CTRL), minimally conscious patients (MCS), and patients diagnosed with unresponsive wakefulness syndrome (UWS). Circles with “+” signs indicate DOC patients who provided evidence of covert consciousness by performing mental imagery tasks in the scanner. White circle, median; blue center line, mean; box limits, upper and lower quartiles; whiskers, 1.5x interquartile range. * *p* < 0.05; *** *p* < 0.001, FDR-corrected across three pairwise comparisons.


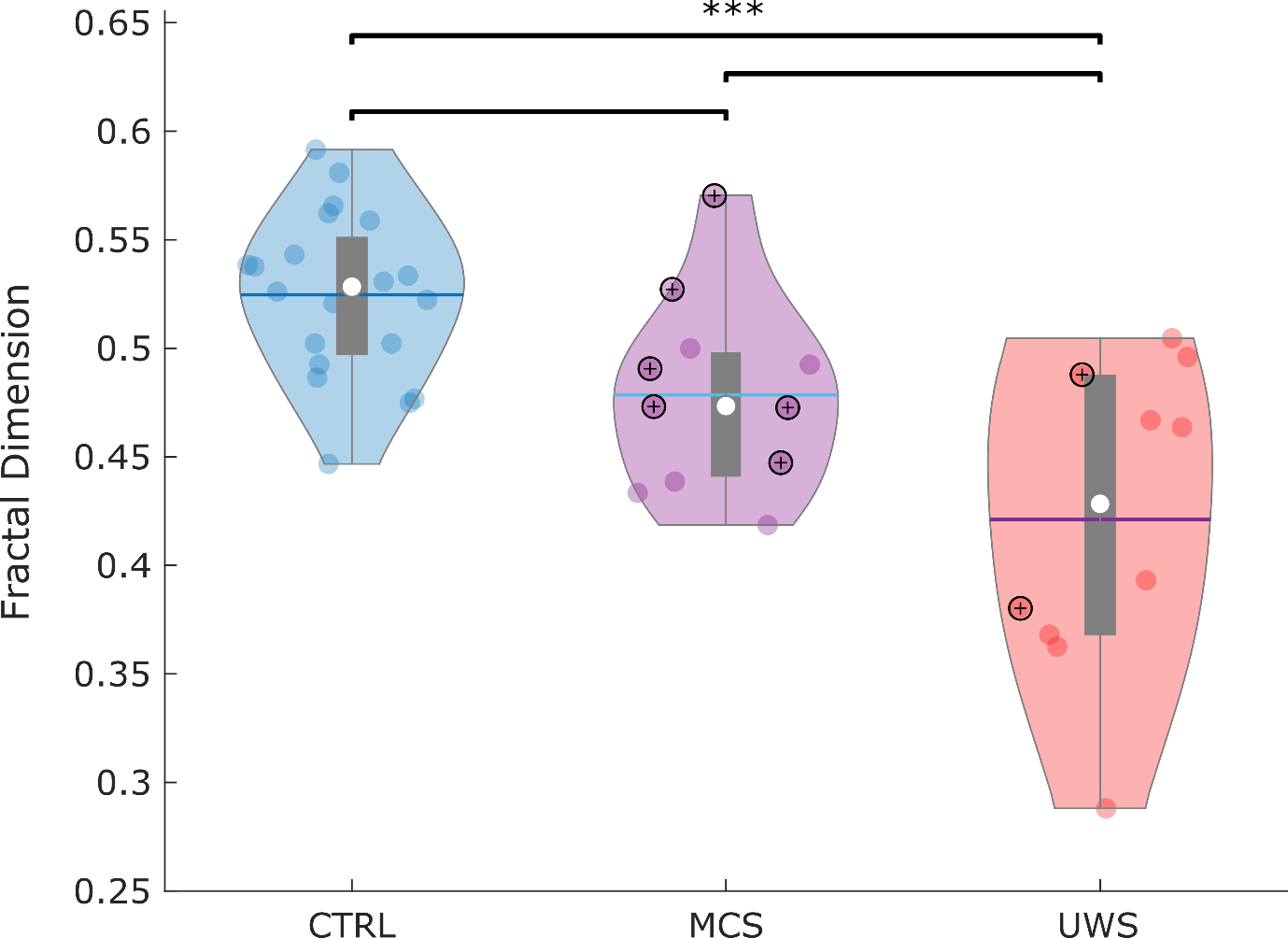


**Supplementary Figure 6. Reduced structural fractal dimension across disorders of consciousness, using the Lausanne parcellation.** Violin plots indicate the distribution of weighted fractal dimension of structural brain networks for healthy controls (CTRL), minimally conscious patients (MCS), and patients diagnosed with unresponsive wakefulness syndrome (UWS). Circles with “+” signs indicate DOC patients who provided evidence of covert consciousness by performing mental imagery tasks in the scanner. White circle, median; blue center line, mean; box limits, upper and lower quartiles; whiskers, 1.5x interquartile range. *** *p* < 0.001, FDR-corrected across three pairwise comparisons.


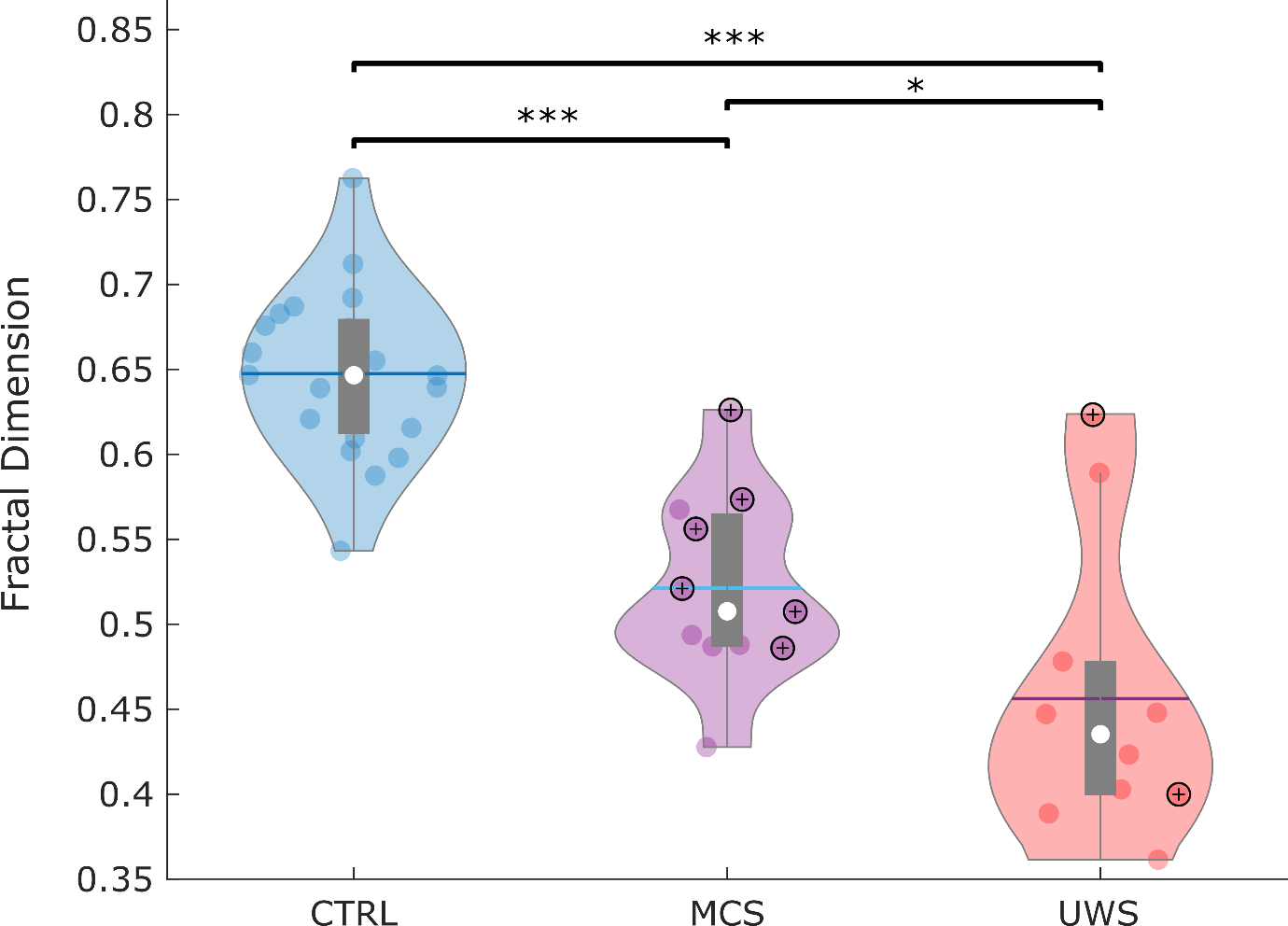


**Supplementary Figure 7. Reduced structural fractal dimension across disorders of consciousness, using the Schaefer scale-400 parcellation.** Violin plots indicate the distribution of weighted fractal dimension of structural brain networks for healthy controls (CTRL), minimally conscious patients (MCS), and patients diagnosed with unresponsive wakefulness syndrome (UWS). Circles with “+” signs indicate DOC patients who provided evidence of covert consciousness by performing mental imagery tasks in the scanner. White circle, median; blue center line, mean; box limits, upper and lower quartiles; whiskers, 1.5x interquartile range. * *p* < 0.05; *** *p* < 0.001, FDR-corrected across three pairwise comparisons.


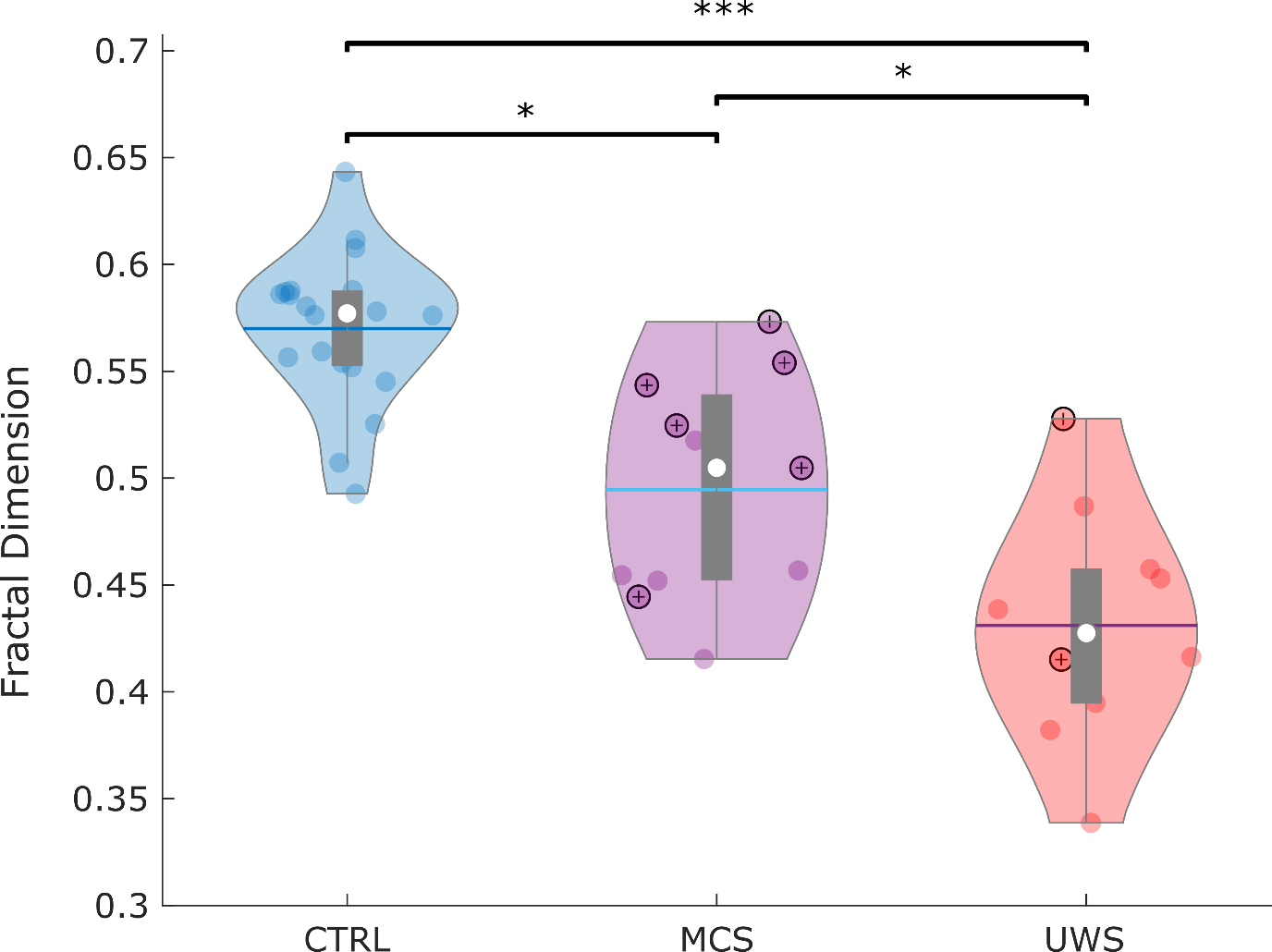


**Supplementary Figure 8. Reduced structural fractal dimension across disorders of consciousness, using binarized edges.** Violin plots indicate the distribution of binary fractal dimension of structural brain networks for healthy controls (CTRL), minimally conscious patients (MCS), and patients diagnosed with unresponsive wakefulness syndrome (UWS). Circles with “+” signs indicate DOC patients who provided evidence of covert consciousness by performing mental imagery tasks in the scanner. White circle, median; blue center line, mean; box limits, upper and lower quartiles; whiskers, 1.5x interquartile range. * *p* < 0.05; *** *p* < 0.001, FDR-corrected across three pairwise comparisons.


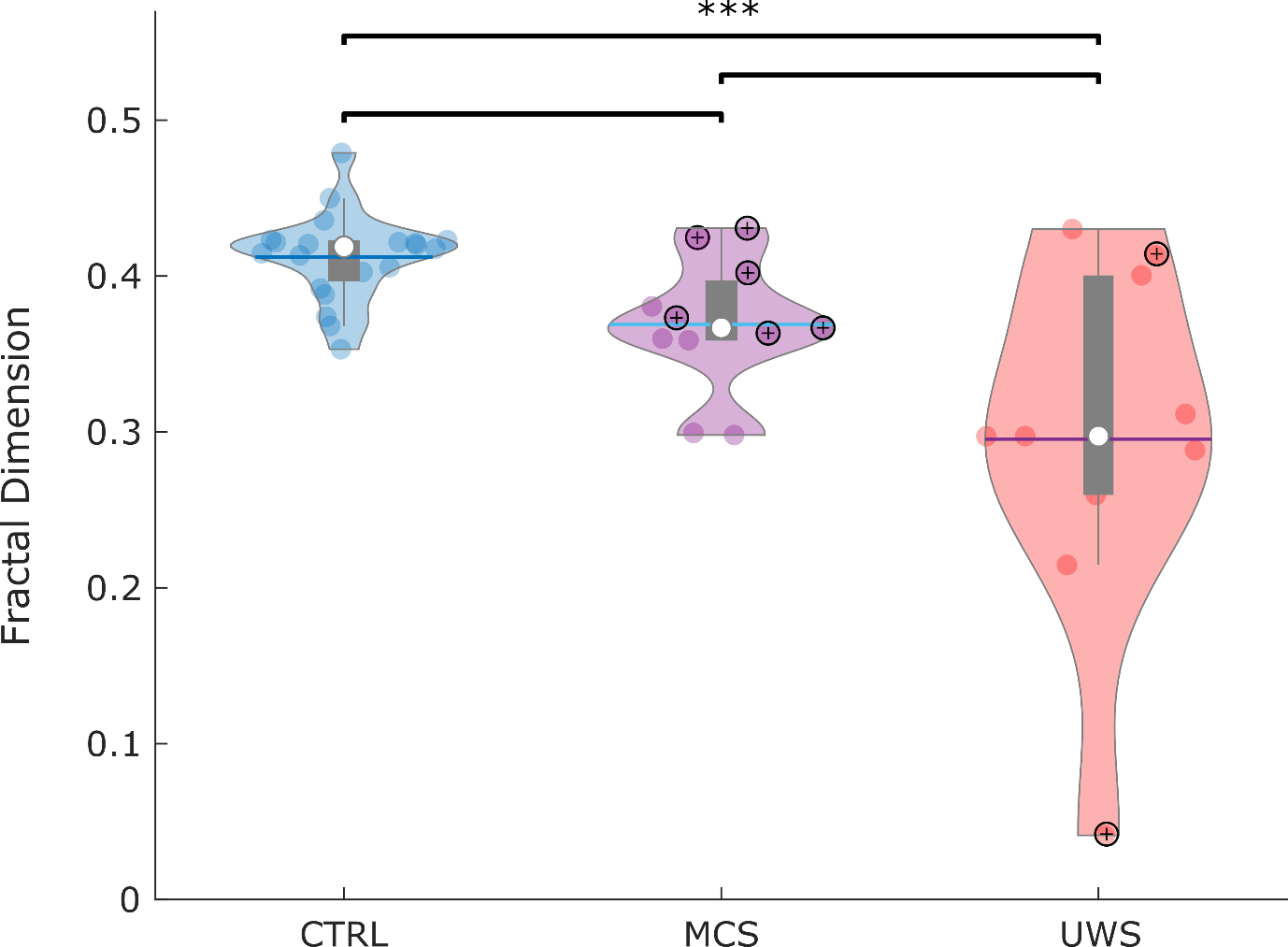


**Supplementary Figure 9. Reduced structural fractal dimension across disorders of consciousness, using alternative tractography.** Violin plots indicate the distribution of weighted fractal dimension of structural brain networks for healthy controls (CTRL), minimally conscious patients (MCS), and patients diagnosed with unresponsive wakefulness syndrome (UWS). Circles with “+” signs indicate DOC patients who provided evidence of covert consciousness by performing mental imagery tasks in the scanner. White circle, median; blue center line, mean; box limits, upper and lower quartiles; whiskers, 1.5x interquartile range. *** *p* < 0.001, FDR-corrected across three pairwise comparisons.

## Supplementary Tables

**Supplementary Table 1. Statistical comparisons of weighted fractal dimension between patients with traumatic (TBI) and anoxic (ANOX) brain injury.**

| Contrast | Estimate | SE | tStat | EffSize | pVal |
| --- | --- | --- | --- | --- | --- |
| TBI vs ANOX | -0.061 | 0.030 | -2.030 | 0.443 | 0.058 |

**Supplementary Table 2.** **Statistical comparisons of weighted fractal dimension between pairs of conditions, using the Schaefer-1000 parcellation, using permutation testing without including additional covariates.**

| Contrast | Mean1 | Mean2 | SD1 | SD2 | tStat | df | EffSize | pVal |
| --- | --- | --- | --- | --- | --- | --- | --- | --- |
| CTRL vs MCS | 0.473 | 0.416 | 0.032 | 0.049 | -3.908 | 29 | -1.429 | 0.001 |
| CTRL vs UWS | 0.473 | 0.348 | 0.032 | 0.063 | -7.258 | 28 | -2.735 | <0.001 |
| MCS vs UWS | 0.416 | 0.348 | 0.049 | 0.063 | -2.765 | 19 | -1.160 | 0.012 |
| FMRI+ vs FMRI- | 0.419 | 0.361 | 0.068 | 0.055 | -2.122 | 19 | -0.915 | 0.047 |

**Supplementary Table 3. Statistical comparisons of weighted fractal dimension between pairs of conditions, using the Lausanne parcellation.**

| Contrast | Estimate | SE | tStat | EffSize | pVal |
| --- | --- | --- | --- | --- | --- |
| CTRL vs MCS | -0.038 | 0.019 | -1.967 | -0.353 | 0.060 |
| CTRL vs UWS | -0.117 | 0.025 | -4.716 | -0.861 | <0.001 |
| MCS vs UWS | -0.047 | 0.026 | -1.817 | -0.397 | 0.087 |
| FMRI+ vs FMRI- | -0.049 | 0.023 | -2.120 | -0.463 | 0.049 |

**Supplementary Table 4. Statistical comparisons of weighted fractal dimension between pairs of conditions, using the Schaefer-400 parcellation.**

| Contrast | Estimate | SE | tStat | EffSize | pVal |
| --- | --- | --- | --- | --- | --- |
| CTRL vs MCS | -0.101 | 0.025 | -4.107 | -0.738 | <0.001 |
| CTRL vs UWS | -0.216 | 0.029 | -7.477 | -1.365 | <0.001 |
| MCS vs UWS | -0.071 | 0.030 | -2.382 | -0.520 | 0.029 |
| FMRI+ vs FMRI- | -0.081 | 0.030 | -2.690 | -0.587 | 0.015 |

**Supplementary Table 5. Statistical comparisons of binary fractal dimension between pairs of conditions, using the Schaefer-1000 parcellation.**

| Contrast | Estimate | SE | tStat | EffSize | pVal |
| --- | --- | --- | --- | --- | --- |
| CTRL vs MCS | -0.060 | 0.021 | -2.792 | -0.501 | 0.010 |
| CTRL vs UWS | -0.169 | 0.023 | -7.283 | -1.330 | <0.001 |
| MCS vs UWS | -0.063 | 0.028 | -2.229 | -0.486 | 0.040 |
| FMRI+ vs FMRI- | -0.077 | 0.023 | -3.350 | -0.731 | 0.004 |

**Supplementary Table 6. Statistical comparisons of weighted fractal dimension between pairs of conditions, using the Schaefer-1000 parcellation with alternative tractography.**

| Contrast | Estimate | SE | tStat | EffSize | pVal |
| --- | --- | --- | --- | --- | --- |
| CTRL vs MCS | -0.029 | 0.016 | -1.756 | -0.315 | 0.090 |
| CTRL vs UWS | -0.131 | 0.024 | -5.384 | -0.983 | <0.001 |
| MCS vs UWS | -0.067 | 0.037 | -1.791 | -0.391 | 0.091 |
| FMRI+ vs FMRI- | -0.078 | 0.033 | -2.343 | -0.511 | 0.032 |
| FMRI+ vs FMRI- (no outlier) | -0.079 | 0.024 | -3.249 | -0.726 | 0.005 |
